# Genomics, transcriptomics, and laboratory experiments link bioconvection to nitrogen fixation

**DOI:** 10.3389/fmicb.2026.1760084

**Published:** 2026-04-02

**Authors:** Francesco Di Nezio, Juan Diaz-Miyar, Antoine Buetti-Dinh, Samuele Roman, Tommaso Fava, Daniele Paganini, Nicola Storelli

**Affiliations:** 1 Anoxic Aquatic Systems Group, Institute of Microbiology, Department of Environment, Constructions and Design, University of Applied Sciences and Arts of Southern Switzerland (SUPSI), Mendrisio, Switzerland; 2 Water Research Institute (IRSA), National Research Council of Italy (CNR), Verbania, Italy; 3 Alpine Biology Center Foundation, Piora, Switzerland; 4 Microbiology Unit, Department of Plant Sciences, University of Geneva (UNIGE), Geneva, Switzerland

**Keywords:** euxinic bottom zone, genomes, meromictic Lake Cadagno, modern analogs, nitrogen fixation, phototrophic sulfur bacteria

## Abstract

**Introduction:**

Lake Cadagno is a meromictic lake characterized by a stable euxinic chemocline that hosts a diverse community of anoxygenic phototrophic sulfur bacteria, among the earliest photosynthetic organisms on Earth. These microorganisms are key to understanding the evolution of photosynthesis; however, due to the rarity of permanently anoxic environments, their genetic and ecophysiological traits remain poorly characterized.

**Methods:**

We generated four high-quality genomes (>93% completeness, < 2% contamination), including two purple sulfur bacteria (PSB; *Chromatium okenii* LaCa and *Thiodictyon syntrophicum* Cad16T) and two green sulfur bacteria (GSB; *Chlorobium phaeobacteroides* 1VII D7 and *Chlorobium clathratiforme* Cad4DE). Using an improved *C. okenii* genome, we analyzed chemocline transcriptomes under conditions with and without bioconvection. Nitrogen fixation potential was assessed through comparative genomic analyses of nif gene content and organization, complemented by laboratory growth experiments under nitrogen-limited conditions.

**Results:**

Nitrogen fixation (nif) genes were significantly upregulated in the chemocline, particularly in September, indicating a potential link between nitrogen fixation and bioconvection. Comparative genomic analyses revealed a higher abundance and diversity of nif genes in PSBs than in GSBs. Laboratory experiments demonstrated that PSBs (*C. okenii, T. syntrophicum*) and the GSB *C. phaeobacteroides* can grow using atmospheric nitrogen as the sole nitrogen source. Light intensity had minimal effects on overall biomass yield but influenced growth rates, while GSBs exhibited reduced performance relative to PSBs under nitrogen limitation.

**Discussion:**

Collectively, genomic, transcriptomic, and experimental evidence confirms active nitrogen fixation in dominant phototrophic sulfur bacteria of Lake Cadagno. The upregulation of nif genes and their association with bioconvection suggest a functional coupling between nitrogen cycling and physical mixing processes, potentially mediated by *C. okenii*. These findings provide new insights into the ecological role of anoxygenic phototrophs in stratified anoxic systems and their contribution to biogeochemical cycling.

## Introduction

Life first emerged on Earth approximately 3.5–4.0 billion years ago, during the Archean eon, marking a crucial turning point in our planet's history (Van Wieren, 2021). Although today's biosphere is largely oxic, early life forms thrived in completely anoxic conditions. These primitive organisms initiated modern biogeochemical cycles through photosynthetic processes that ultimately triggered the “Great Oxidation Event,” a transformative shift that enabled the evolution of aerobic metabolic processes (Fischer et al., 2016; Demoulin et al., 2019; Uveges et al., 2023). The transition to aerobic metabolism conferred a substantial energetic advantage, as well as modifying the atmosphere by creating a protective ozone layer, facilitating the evolutionary diversification of life even beyond aquatic environments. Today, anoxic environments reminiscent of primordial oceans are extremely rare and typically restricted to “*extreme environments*,” making them difficult to study.

Meromictic lakes provide valuable natural laboratories for studying ancient ecosystems, as their permanent stratification maintains oxygen-depleted layers that support anoxic life forms, effectively representing “*modern analogs*” of early Earth environments (Gulati et al., 2017). The euxinic (i.e., anoxic and sulfidic) or ferruginous (i.e., anoxic and iron-rich) conditions within these lakes provide valuable insights into early microbial metabolic pathways and their potential analogs in extraterrestrial environments, where similar redox gradients may exist (Canfield, 1998; Poulton et al., 2004; Xiong et al., 2019). Microbial life in these ecosystems is often represented by sulfate-reducing bacteria (SRB) and methanogenic archaea typically found in the deepest dark anoxic layers (monimolimnion) and sediments, where they mediate the reduction of sulfur and iron, or methane production. In shallow meromictic lakes, where light penetrates into the oxic-anoxic redox transition zone called chemocline, dense communities of anoxygenic phototrophic purple (PSB) and green (GSB) sulfur bacteria thrive (Overmann and Van Gemerden, 2000). PSB and GSB are considered among the earliest phototrophic lineages to have evolved, retaining metabolic traits that predate oxygenic photosynthesis and thus providing key insights into the evolution of early energy metabolisms under anoxic conditions (Martin et al., 2018).

Lake Cadagno, located in the Swiss Alps, is a well-documented example of crenogenic meromixis, a particular limnological phenomenon driven by the inflow of mineral-rich groundwater (Otz et al., 2003). The euxinic layer, located at approximately 12 meters depth, supports a dense phototrophic bacterial layer (BL) consisting of at least six PSB and two GSB species (Tonolla et al., 2005; Decristophoris et al., 2009; Danza et al., 2018; Di Nezio et al., 2023). These microorganisms are integral to major biogeochemical cycles, particularly in carbon and sulfur cycling, where they contribute to organic carbon fixation and sulfur transformation, influencing the lake's overall ecosystem dynamics (Storelli, 2014; Posth et al., 2017; Luedin et al., 2019b). Recent studies suggest that these bacteria also play a significant role in the nitrogen cycle, suggesting their ability to fix nitrogen, an essential process for sustaining microbial life in nutrient-limited environments (Philippi et al., 2021).

This great biodiversity of anoxygenic phototrophic sulfur bacteria observed in the BL is also reflected in the diverse evolutionary strategies employed to dominate their ecological niche. One such strategy is bioconvection, i.e., the collective motion of microorganisms that can mix water columns up to a meter deep, a phenomenon rarely documented in nature (Sommer et al., 2017). In fact, the motile PSB *Chromatium okenii* swims phototactically thanks to a tuft of flagella, but upon sensing oxygen, it suddenly stops movement, increasing local water density. This denser water then sinks due to gravity, dragging the bacteria down with it. This collective mixing enhances access to light and nutrients, redistributes metabolic by-products, and helps maintain cells within optimal redox and irradiance conditions, thereby conferring a clear ecological advantage in a steeply stratified environment (Sepúlveda Steiner et al., 2021; Di Nezio et al., 2023). However, studying and cultivating these microorganisms under controlled laboratory conditions remains challenging due to the difficulty of reproducing key environmental parameters such as redox gradients, light availability, and sulfide concentrations. Laboratory-grown *C. okenii* exhibits marked phenotypic differences compared to its natural counterparts, underscoring the limitations of traditional cultivation methods and the importance of *in situ* studies (Di Nezio et al., 2024).

Recent advancements in sequencing technologies, such as single-cell DNA/RNA sequencing, metagenomics and transcriptomics, have provided unprecedented insights into the composition and functional potential of microbial communities without the need for cultivation (Emerson et al., 2008; Rinke et al., 2013). These approaches have facilitated the discovery of novel metabolic pathways and microbial interactions, revealing the adaptive strategies employed by anaerobic microorganisms in response to environmental stressors (Baker et al., 2013; Mackelprang et al., 2016; Barua et al., 2017). However, transcriptomic analyses remain challenging, particularly due to the absence of comprehensive reference databases, which complicate accurate annotation and differentiation between known and potentially novel genes (Emerson et al., 2008; Kopcakova et al., 2014; Choi et al., 2016). While *de novo* assembly methods offer a viable solution, they also introduce additional complexities and potential errors in gene prediction and functional annotation (Baker, 2012; Liao et al., 2019).

In this study, we sequenced the genomes of the four dominant phototrophic species in the BL, which represent more than 80% of the phototrophic sulfur bacteria cells (Di Nezio et al., 2023; Storelli et al., 2025). These high-quality genomes expand the genetic repertoire of anoxygenic phototrophic sulfur bacteria and enable a detailed investigation of the bioconvection process mediated by the fully sequenced purple sulfur bacterium *Chromatium okenii* through transcriptomic analyses. Gene expression in *C. okenii* was examined directly in the lake environment to minimize experimental artifacts by comparing BL transcriptomes collected during periods with active bioconvection in summer (July) and periods without bioconvection in autumn (September). Finally, the capacity for growth under nitrogen-free conditions was assessed experimentally in the laboratory, providing evidence consistent with the potential for PSB, alongside GSB, to contribute to nitrogen fixation.

## Material and methods

### Study site and sampling

Lake Cadagno is located in the Piora Valley at 1921 m above sea level, in the southern Swiss Alps (46 °33′ N, 8 °43′ E, depth approximately 21 m). In addition to surface water tributaries, the lake receives inflows from sublacustrine springs, which supply high-density water that flows through gypsum-rich (CaSO_4_) dolomite rock (CaMg(CO_3_)_2_). The interplay of high salinity and low temperature maintains a dense, anoxic monimolimnion that remains stably stratified beneath the clear and oxygenated mixolimnion originating from the granitic zone. The chemocline at approximately 12 m depth harbors the dense phototrophic bacterial layer (BL), which was the main source of samples for genome and transcriptome analyses. The conductivity in the lower layer (monimolimnion) ranges between 0.20 and 0.25 mS cm^−1^ (Supplementary Figure S3, orange line), mainly due to the presence of carbonates (${\text{HCO}}_{3}^{-}$ up to 50 mg L^−1^) and sulfates (${\text{SO}}_{4}^{-2}$ up to 200 mg L^−1^) originating from dolomite (Del Don et al., 2001).

Physicochemical parameters of the water column were determined using a multiparameter probe (CTD115M, Sea & Sun Technology, Trappenkamp, Germany) equipped with pressure (bar), temperature (°C), conductivity (mS cm^−1^), dissolved oxygen (mg L^−1^), and turbidity (Formazine Turbidity Unit, FTU) sensors. Moreover, the CTD is further equipped with a photosynthetically active radiation (PAR, 400–700 nm) sensor (LI-COR Biosciences, Lincoln, NE, USA), detecting the spectral range (wave band) of solar radiation from 400 to 700 nm used by photosynthetic organisms in the process of photosynthesis, and a phycocyanin fluorescence (BGAPC) sensor (Turner Designs, San José, CA, USA). Different water samples were taken at the appropriate depths and analyzed chemically (50 mL and 12 mL with 5% zinc acetate) and biologically (1.5 mL) as described in Di Nezio et al. (2021). The water column profiles measured during the sampling campaigns for transcriptomic analyses on 16 July 2020 and 17 September 2020 are presented in the Supplementary Figure S3.

### Isolation and growth conditions of anoxygenic phototrophic sulfur bacteria

The different strains of anoxygenic sulfur bacteria were monitored, isolated from Lake Cadagno, and cultivated in the laboratory over the past 20 years (see Table 1). From this culture collection, representative strains were selected for genome sequencing and physiological testing, including nitrogen fixation experiments. Phototrophic sulfur bacteria were grown in Pfennig's medium (Trüper, 1970) type I for PSB and type II for GSB both of which containing 0.25 g L^−1^ of KH_2_PO_4_, 0.34 g L^−1^ of NH_4_Cl, 0.5 g L^−1^ of MgSO_4_ 7H_2_O, 0.25 g L^−1^ of CaCl_2_ 2H_2_O, 0.34 g L^−1^ of KCl, 1.5 g L^−1^ of NaHCO_3_, in addition to different concentrations of carbonate, sulfide, and solutions of vitamins and trace elements, as shown in detail in the specifications outlined in the previous studies referenced in Table 1.

**Table 1:** List of anoxic phototrophic sulfur bacteria fully sequenced in this study, isolated in the past and maintained in pure cultures in our laboratory (References).

Type	Species	References
PSB	*Thiodictyon syntrophicum* Cad16^T^	Peduzzi et al., 2012
PSB	*Chromatium okenii* LaCa	Luedin et al., 2019a
GSB	*Chlorobium phaeobacteroides* 1VII D7	Di Nezio et al., 2021
GSB	*Chlorobium clathratiforme* Cad4DE	Gregersen et al., 2009

PSB, purple sulfur bacteria; GSB, green sulfur bacteria.

### Nitrogen fixation growth assays

To assess growth under nitrogen-replete (Standard) and nitrogen-depleted (No NH_4_Cl) conditions, cultures of PSB *C. okenii* LaCa and *T. syntrophicum* Cad16^T^, were incubated in Pfennig medium I (with 0.34 g L^−1^ of NH_4_Cl) and in a modified one without NH_4_Cl (with 0.34 g L^−1^ of NaCl). GSB *C. phaeobacteroides* 1VII D7 was used as a positive control. Ammonium concentrations were measured in all media prior to inoculation and at the end of the incubation period by photometric measurement using the Spectroquant Merck Ammonium Test Kit (1.00683: 2.0–150 mg L^−1^ (NH_4_-N), 2.6-193 mg L^−1^ (${\text{NH}}_{4}^{+}$)). Concentrations below the detection limit of the assay were reported as zero.

Cultures were incubated under anoxic conditions at two light intensities 4.0 μE m^−2^ s^−1^, simulating the conditions of the lake, and 40.0 μE m^−2^ s^−1^, representing the laboratory light regime. Both incubation settings followed a 16/8-h light-dark photoperiod. All cultures were done in triplicate and growth was monitored over a 12-day incubation period. Growth trajectories were analyzed using linear mixed-effects models implemented in R (version 4.5.0), with time, medium (N^+^ vs. N^−^), and light intensity included as fixed effects and biological replicate as a random effect. Time was modeled using natural splines to accommodate non-linear growth dynamics, as implemented in the *splines* package (Wang and Yan, 2021). Mixed-effects models were fitted using the *lme4* package (Bates et al., 2015), and statistical significance of fixed effects was assessed using Satterthwaite's approximation as implemented in *lmerTest* (Kuznetsova et al., 2017). Analyses were conducted separately for each strain. Final biomass was analyzed using linear mixed-effects models with medium and light as fixed effects and replicate as a random effect.

### Flow cytometer

Flow cytometry (FCM) was used to monitor the growth and purity of the cultures. The analysis was conducted with a BD Accuri C6 flow cytometer equipped with two lasers (488 nm and 640 nm), dispersion and fluorescence detectors. Two parameters were measured: FSC (particle size) and SSC (internal granularity). To identify photosynthetic bacteria, an FSC-H threshold of 2,000 was applied to exclude debris and abiotic particles, followed by an FL3-A threshold > 1,100 to select cells with autofluorescence from chlorophyll or bacteriochlorophyll. The analysis was limited to 50 μL per sample, with dilution if necessary to not exceed 1,000 events mL^−1^, as previously shown (Danza et al., 2017; Di Nezio et al., 2021).

### DNA extraction and sequencing

After being cultivated in the laboratory (see previous point “*Isolation and growth conditions of anoxygenic phototrophic sulfur bacteria*“), all the samples were filtered with a polycarbonate filter (Isopore 0.2 μm PC membrane, 25 mm diameter) using a vacuum pump (Vacuubrand GmbH Co. KG, Wertheim, Germany) connected to the filtration ramp (Pall Corporation, New York, NY, USA) until the filter was completely clogged (aprox. 5–10 mL). Genomic DNA was extracted with the phenol chloroform extraction protocol provide by Thermo-Fisher scientific (standard protocol). Genomes were sequenced by Fasteris (GeneSupport SA) using PacBio SMRT Hi-Fi sequencing on a Sequel IIe system. FASTQ files were QC-checked using FastQC (v.0.11.9) and deemed of good quality (Simon Andrews, 2010).

### Genome assembly and annotation

*De novo* assembly was performed using Flye (v2.9.4) and polished using Circlator (v1.5.5) to remove repetitive regions, attempt chromosome circularization, and set the start coordinate at the *dna*A gene (Hunt et al., 2015; Kolmogorov et al., 2019). All individual assemblies were manually reviewed to assess the quality of the identified contigs and remove artifacts from the sequencing and assembly process, including the removal of contigs smaller than 10 kbp. All *de novo* assemblies were checked for contamination and completeness using CheckM (v1.2.2) and BUSCO (v5.8.2_cv1; Parks et al., 2015; Tegenfeldt et al., 2025). Genes were annotated using the NCBI Prokaryotic Genome Annotation Pipeline (v2025-05-06.build7983; Tatusova et al., 2016). Functional annotation of genomes, including Clusters for Orthologous Groups (COG) category assignment, was performed using eggnog-mapper (v2.1.12; Cantalapiedra et al., 2021). All assembled genomes and raw sequences were submitted to ENA and are available under BioProject PRJEB110913.

### Phylogenetic analysis

Average Nucleotide Identity (ANI) was calculated with FastANI (v1.34; Jain et al., 2018). Representative full length 16S rRNA sequences were retrieved from the Bacterial 16S rRNA RefSeq Targeted loci project (PRJNA33175) or extracted from the *de novo* assemblies using R Biostrings (v2.76.0). Complete genome assemblies for our bacteria of interest were retrieved from NCBI Datasets, comprising 11 GSB and 22 PSB genomes. All sequence analysis were performed separately for the two bacterial lineages (GSB and PSB).

Maximum likelihood (ML) phylogenetic trees were constructed using two distinct approaches: one based on bacterial 16S rRNA sequences, and another based on the amino acid sequences of 100 concatenated single-copy orthologs selected randomly. Single-copy orthologous proteins were identified using OrthoFinder (Emms and Kelly, 2019). Both datasets were aligned using MUSCLE (v3.8.31), and poorly aligned regions were trimmed using trimAl using the *automate1* option (Edgar, 2004; Capella-Gutiérrez et al., 2009). ML tree inference was conducted using IQ-TREE 3 with 1000 ultrafast bootstrap replicates and 1000 aLRT tests to assess branch support (Hoang et al., 2018). ModelFinder was employed to automatically select the best-fit substitution model for nucleotide sequences (Kalyaanamoorthy et al., 2017). For the protein dataset, a partitioned model approach was applied to determine the best-fit amino acid substitution model for each gene partition (Chernomor et al., 2016). Tree visualizations were prepared using iTOL v7 (Letunic and Bork, 2024).

### Transcriptomics: RNA extraction and analysis

To ensure enough RNA concentration, we first cultivated *C. okenii* LaCa in the laboratory and then placed them in the BL zone at a second stage using 50 cm vertical long dialysis bags (inflated diameter of 62.8 mm; Karl Roth GmbH Co. KG, Karlsruhe, Germany), These bags allow small molecules (< 20 kDa) to pass through but isolate *C. okenii* LaCa from other microorganisms in the BL, as already shown in previous studies (Storelli et al., 2013; Di Nezio et al., 2021). The samples enclosed in the dialysis bags were incubated for 1 month prior to RNA analysis, so that they had time to adapt to the environmental conditions.

*C. okenii* LaCa was isolated using filters for transcriptomic analysis, which were soaked in RNA later (Qiagen, Hilden, Germany) for 5 min immediately after filtration and then frozen at −20 °C, from three different dialysis bags, the first time on July 16, 2020 (with bioconvection; Supplementary Figure S3A) and the second time on September 17, 2020 (no bioconvection; Supplementary Figure S3B). RNA was extracted using the RNeasy plus Universal mini kit (Qiagen) following the protocol “Purification of total RNA Using the RNeasy Plus Universal mini kit” for the TissueLyser II, using the complete filter as starting material and using a mixture of glass beads of different sizes 0.1 mm, 0.5 mm and 1.0 mm. DNase treatment was performed using Ambion^®^ Turbo DNA-freeTM kit (Thermo Fisher Scientific, Waltham, MA, USA) following the manufacturer's instructions. Quantification of RNA was carried out with the QubitTM RNA HS Assay kit (Thermo Fisher Scientific) using a volume of 1.0 mL. Nanodrop absorbance ratios 260/280 nm and 260/230 nm were measured to check for impurities.

Complementary DNA (cDNA) for sequencing was prepared using the PCR-cDNA Barcoding Kit (SQK-PCB109; Oxford Nanopore Technologies, Oxford, UK) following the manufacturer's instructions. 50–100 fmol in 11.0 mL of reverse transcribed DNA were used for Oxford Nanopore Technologies (ONT) library preparation according to manufacturer instructions (Kit SQK-PCB109) and sequencing was performed with an ONT R9.4 flow cell. Quality Control (QC) metrics of the RNA sequencing with MinION of lake dialysis bag samples in July and September (Supplementary Table S3).

Basecalling was performed on raw FAST5 files using Guppy (v4.5.2), adapter removal was performed using pychopper (v2.5.0), followed by one step of poly-A removal using cutadapt (v4.6). Ribosomal reads were removed using RiboDetector (v0.3.1) prior to transcript quantification with oarfish (v0.6.5) using the *de novo C. okenii* assembly presented in this study (Deng et al., 2022; Zare Jousheghani et al., 2025). Reads mapping to RNA genes were excluded from the analysis. All subsequent analysis were run on R (v.4.5.0) using packages in the tidyverse (v2.0.0) for data manipulation and visualization. Differential expression analysis was run on the resulting count files using DESeq2 (v1.48.0) with apeGLM for LFC shrinkage (v1.30.0; Love et al., 2014; Zhu et al., 2019). Genes with an absolute log fold change |logFC| > 1, base Mean > 5.7 counts, and adjusted p-value < 0.05 were defined as differentially expressed.

Gene Set Enrichment Analysis (GSEA) was performed using clusterProfiler (v4.16.0) and fgsea (1.34.0; Yu et al., 2012; Korotkevich et al., 2021). The Gene Ontology (GO) database was created using AnnotationForge (v1.50.0) from Bioconductor.

## Results

### High-quality genome assemblies

We successfully assembled and annotated four bacterial genomes isolated from Lake Cadagno, two purple sulfur bacteria (PSB, family *Chromatiaceae*), and two green sulfur bacteria (GSB, family *Chlorobiaceae*). All genomes were sequenced using PacBio HiFi and assembled with Flye, resulting in high quality assemblies with high completeness (>93%) and low contamination (< 2%), as assessed by CheckM and BUSCO (Table 2; further details in Supplementary Table S1). The PSB strains sequenced include *Chromatium okenii* LaCa and *Thiodictyon syntrophicum* Cad16^*T*^. The GSB are *Chlorobium phaeobacteroides* 1VII D7 and *Chlorobium clathratiforme* Cad4DE. Genome sizes ranged from approximately 3.0–7.7 Mbp, with contig numbers between 1 and 4. N50 values were consistently high, reflecting the contiguity of the assemblies (Table 2).

**Table 2:** Genome assembly characteristics.

Organism	Type	Genome size (bp)	Contig number	GC content (%)	N50 (Mbp)	Cov.	Complet. (%)	Contam. (%)	ENA accession
*Chromatium okenii* LaCa	PSB	3′137′866	2	49.96	3.127	194x	93.51	0.28	**GCA_982375535.1**
*Thiodictyon syntrophicum* Cad16^T^	PSB	7′736′645	3	66.22	6.835	24x	99.25	0.32	**GCA_982375505.1**
*Chlorobium phaeobacteroides* 1VII D7	GSB	3′104′529	1	48.24	3.105	218x	93.37	2.03	**GCA_982375525.1**
*Chlorobium clathratiforme* Cad4DE	GSB	3′007′024	1	48.05	3.007	42x	97.64	0.46	**GCA_982375515.1**

All strains were sequenced with PacBio HiFi long reads and assembled with Flye. Completeness and contamination values were determined by CheckM.

Annotation revealed between 2,849 and 6,743 protein-coding genes per genome, with varying numbers of rRNA operons, tRNAs, and CRISPR arrays (Table 3). Notably, *T. syntrophicum* presented the largest genome and highest gene count, while the other genomes were approximately half its size. Cluster of Orthologous genes (COG) functional classification of genes from the complete dataset can be seen in Supplementary Table S2.

**Table 3:** Genome annotation summary.

Organism	Type	Protein-coding genes	rRNA genes (5S, 16S, and 23S)	tRNAs	ncRNAs	Pseudogenes	CRISPR arrays
*Chromatium okenii* LaCa	PSB	2,849	9 (3, 3, 3)	49	4	36	3
*Thiodictyon syntrophicum* Cad16^T^	PSB	6,743	6 (2, 2, 2)	50	4	128	5
*Chlorobium phaeobacteroides* 1VII D7	GSB	2,841	6 (2, 2, 2)	47	3	62	4
*Chlorobium clathratiforme* Cad4DE	GSB	2,933	6 (2, 2, 2)	47	3	83	3

**Figure 1:**
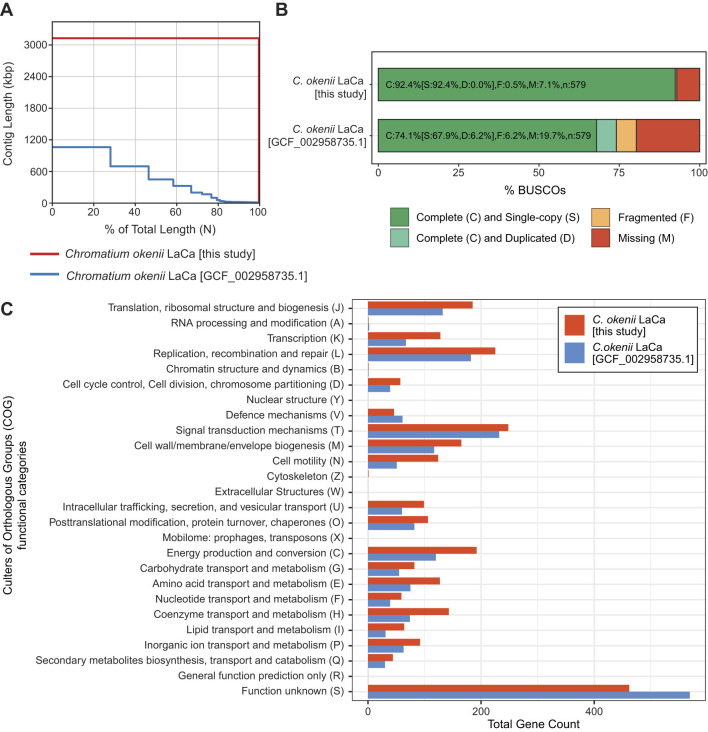
Comparison of the *Chromatium okenii* LaCa genome assembly generated in this study with the previously published assembly (GCF_002958735.1; Luedin et al., 2019a). **(A)** Contig length (kbp) plotted against the cumulative percentage of total assembly length. **(B)** BUSCO completeness metrics. **(C)** Number of genes assigned to each COG functional category. In all panels, the assembly from this study is shown in red and the previously published assembly in blue.

Figure 1 shows the assembly for the PSB *C. okenii* LaCa strain represents a major improvement over the previously published assembly (GCF_002958735.1), now consisting of a single circular chromosome and a secondary circular contig (approx. 10 kbp). This contig did not map the main chromosome and contained a DNA polymerase, a recombinase and a transcription regulator apart from other hypothetical proteins.

Genome completeness assessment using BUSCO revealed a slightly reduced completeness for the *C. okenii* genome compared to the Chromatiaceae lineage, with 7.1% of expected single-copy orthologs missing (Supplementary Table S1). Among these, the canonical replication initiator gene *dna*A was notably absent from all assemblies existing for this species. To assess whether this absence reflects an artifact or a lineage-specific feature, we performed an orthology-based comparative analysis across 23 complete Chromatiaceae genomes using OrthoFinder (see material and methods section). The orthogroup containing *dna*A was absent in all *C. okenii* assemblies available and in only one other Chromatiaceae genome, whereas orthogroups encoding other core components of the replication machinery, such as *dna*B, *dna*N and the gyrases *gyr*A and *gyr*B, were conserved across all genomes analyzed.

**Figure 2:**
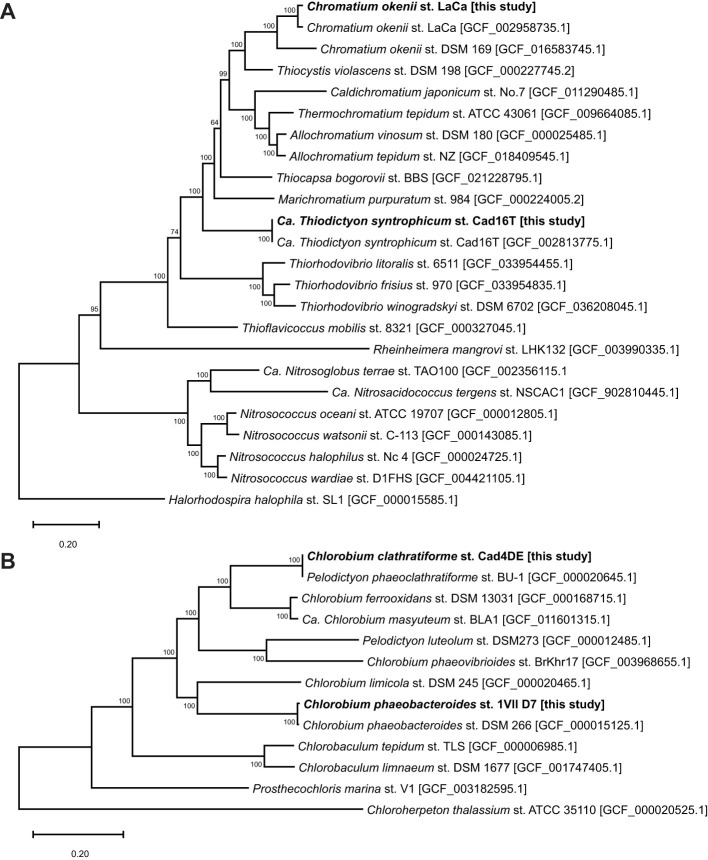
Phylogenetic relationship of the four bacteria within all publicly available complete Genomes of closely related species. The maximum likelihood consensus tree was constructed from 100 single-copy orthologs randomly selected. Bootstrap support values are shown for nodes with support higher than 70%. **(A)** Phylogenetic tree for orthologous sequences of Chromatiales genomes. **(B)** Phylogenetic three for orthologous sequences of Chlorobiales genomes. Ca., Candidatus; st., strain.

The genome of *Thiodictyon syntrophicum* Cad16^T^ closely matched the previously published assembly (GCF_002813775.1), confirming its identity and genomic stability nearly 10 years later. *Chlorobium phaeobacteroides* 1VII D7 was assembled into a single contig with 2,851 genes and showed 98.789% ANI with the DSM 266 strain (GCF_000015125.1), previously isolated from meromictic Lake Blankvann in Norway. The high ANI and nearly identical 16S rRNA sequences (99.87% of blast identity) confirm that both strains belong to the same species. *Chlorobium clathratiforme* Cad4DE was assembled into a complete genome for the first time. This strain, synonymous with *Pelodictyon clathratiforme* and *Pelodictyon phaeoclathratiforme*, shares 99.996% ANI with the BU-1/DSM 5477 strain, originally isolated from the monimolimnion of Lake Buchensee, Germany. This represents the first complete genome under the name *Chlorobium clathratiforme*. The similarities between the old and new genomes are evident both at the orthologous protein sequence level (Figure 2) and at the 16S rRNA level, as illustrated by the phylogenetic tree (Supplementary Figure S1).

### Bioconvection: seasonal changes in gene expression

To explore the effect of bioconvection on *C. okenii* physiology, we performed exploratory RNA sequencing *in situ* on pure cultures using dialysis bags at two different times: in July (bioconvection active) and in September (inactive). Differential expression analysis between July and September samples identified a total of 91 differentially expressed genes (DEGs, 28 downregulated and 68 upregulated), from a total of 770 protein-coding genes detected in the experiment (Figure 2A and Supplementary Table S4). Gene Set Enrichment Analysis (GSEA) was performed to investigate the biological significance of the 91 DEGs (Supplementary Figure S4).

**Figure 3:**
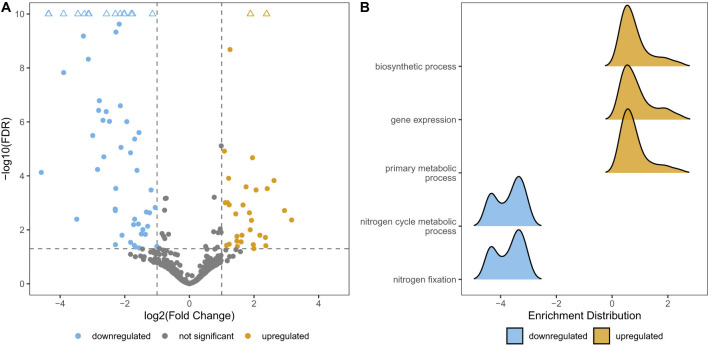
Differential analysis of gene expression of C. okenii cultures in dialysis bags between July and September. **(A)** Volcano plot of differentially expressed genes between July and September, with July taken as a reference point in terms of gene expression level. FDR values are capped to 10–10 and displayed as triangle shapes for visualization purposes. **(B)** Gene set enrichment analysis (GSEA) of Biological Process (BP) terms. Highest-scoring five gene categories are shown. All enriched categories have adjusted *p*-values < 0.0001.

Genes associated with nitrogen fixation (*nif* HDK, *nif* ENB, *nif* T, *nif* V) were strongly downregulated in July, a period characterized by active bioconvection (Figure 3B). We also observed enrichment of Gene Ontology (GO) terms that indicate cell proliferation such as gene expression, biosynthetic process and primary metabolic process (also translation initiation and macromolecule biosynthesis, Supplementary Table S5) coinciding with the seasonal occurrence of bioconvection. Among genes that were enriched in July, we found the chaperonin *gro*EL, *nuo*F, and *nuo*G (involved in oxidoreduction), and the light-harvesting antenna LH1. We also compared daily variations in gene expression between day and night in July, to try to understand why bioconvection persists even without light. The result of the comparison between day and night showed no significant changes in gene expression (data not shown).

### Nitrogen fixation

#### Nitrogen pathway annotation

**Figure 4:**
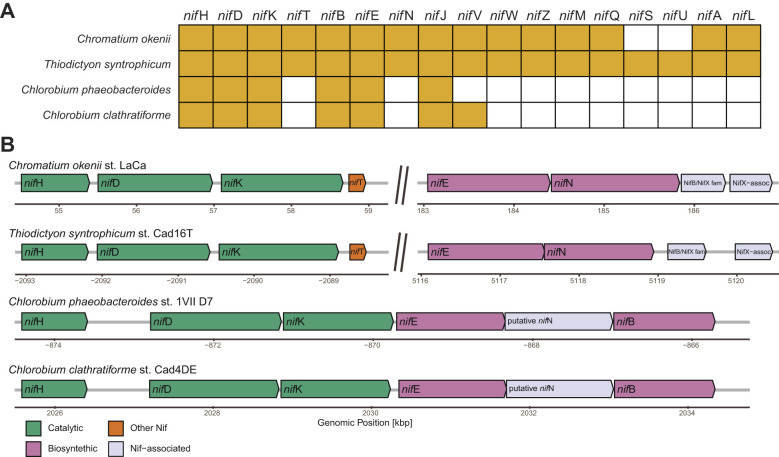
Nitrogen pathway in two PSB and two GSB genomes. **(A)** Presence of the key nif genes in the four genomes sequenced and annotated in this study. **(B)** Operons arrangement in the genomes of the two PSBs and two GSBs considered. Catalytic nif subunits are shown in green while biosynthetic subunits are shown in pink. Genes are grouped in clusters of at least 4 features separated by less than 30 kb.

Nitrogenase (*nif* ) genes are usually found in highly conserved operons and generally have very similar phylogenetic histories. We found the three core nitrogenase components *nif* HDK and the cofactor assembly proteins *nif* ENB in all the assembled genomes (Figure 4).

#### Nitrogen fixation in laboratory

The presence in the genome of the necessary genes for fixing inorganic nitrogen has been experimentally verified in the laboratory. We monitored the growth capacity of PSB *C. okenii* LaCa and *T. syntrophicum* Cad16^T^, as well as GSB *C. phaeobacteroides* 1VII D7, in normal Pfennig medium with a nitrogen source (standard: with NH_4_Cl) and without (no NH_4_Cl: with NaCl). The growth of all phototrophs was monitored at two different light intensities, one similar to environmental conditions (4 μE m^−2^ s^−1^) and the other with a higher intensity similar to laboratory conditions (40 μE m^−2^ s^−1^).

The presence of NH_4_ in the medium before and after bacterial growth was measured to assess: (1) its actual utilization, and (2) the potential production of excess ammonium. Before the growth experiment, a value of 92.0 ± 2 mg L^−1^ was measured in the standard Pfennig medium, while in the other (No NH_4_Cl) medium, the value was 0.0 mg L^−1^. After 12 days of incubation, we measured the ammonium concentrations in all cultures again. In the standard soil, we saw a reduction, with final values of 3.7, 1.8, and 5.6 mg L^−1^ of ammonium for *C. okenii* LaCa, *T. syntrophicum* Cad16^T^, and *C. phaeobacteroides* 1VII D7, respectively. In Pfennig media modified with NaCl instead of NH_4_Cl, we found no trace of ammonium, which therefore remains at 0.0 mg L^−1^ for all cultures even after the experiment.

**Figure 5:**
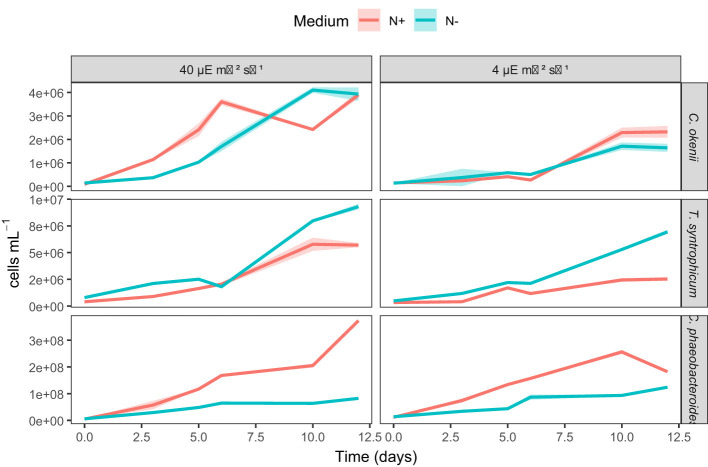
Growth curves with and without nitrogen sources. Graphs showing growth curves for C. okenii LaCa, T. syntrophicum Cad16T and C. phaeobacteroides 1VII D7 under two different light intensities (40 and 4 μE m^−2^ s^−1^) and nitrogen-replete (N^+^) or nitrogen-depleted (N^−^) conditions. Shaded areas indicate ±1 SD. Cell abundance was determined by flow cytometry.

Figure 5 shows that all microorganisms can survive and reproduce under all growth conditions. The two PSB strains showed similar growth in both standard Pfennig medium (with NH_4_Cl) and modified medium (without NH_4_Cl) with only nitrogen in gaseous form (Figure 5, green line). Mixed-effects modeling of growth curves indicated that, in *C. okenii* and *T. syntrophicum*, nitrogen availability and light intensity modulated growth dynamics in a time-dependent manner; however, these effects did not translate into consistent differences in mean growth levels (Supplementary Table S7). In contrast, GSB *C. phaeobacteroides* 1VII D7 showed a reduced growth rate in the absence of a nitrogen source (Figure 5, green line). Despite strain-specific differences in growth trajectories, analyses of final biomass revealed no significant differences between nitrogen-replete and nitrogen-depleted conditions for any of the three strains, nor significant medium × light interactions (Supplementary Table S7). For all microorganisms tested, no significant differences were found at different light intensities.

## Discussion

Our study presents new findings on the genomic and eco-physiological characteristics of anoxygenic phototrophic sulfur bacteria, precursors of modern oxygenic photosynthesis that transformed Earth approximately 3.8 billion years ago (Uveges et al., 2023). High-quality genome assemblies of two PSB species (*Chromatium okenii* LaCa and *Thiodictyon syntrophicum* Cad16^T^) and two GSB species (*Chlorobium phaeobacteroides* 1VII D7 and *C. chlatratiforme* Cad4DE) which characterize the bacterial layer (BL) phototrophic community in meromictic Lake Cadagno, have enhanced our understanding of two key eco-physiological processes: nitrogen fixation and bioconvection (Philippi et al., 2021; Di Nezio et al., 2023). Although nitrogen is essential for all organisms, biological nitrogen fixation only occurs under anoxic conditions and is therefore restricted to a small subset of prokaryotes, including the well-studied GSBs (Halm et al., 2009; Zimmermann et al., 2015) and the emerging PSBs (Philippi et al., 2021). Bioconvection, a phenomenon rarely observed in nature (Sommer et al., 2017; Di Nezio et al., 2023; Sepúlveda Steiner et al., 2023), is still not well understood. Transcriptomic analysis of *C. okenii* LaCa revealed distinct metabolic processes in the presence and absence of bioconvection, showing a negative correlation between periods of active bioconvection and *nif* gene expression.

### Genome assemblies and evolutionary context

The previous genome of the dominant PSB species in Lake Cadagno, *C. okenii*, which can generate bioconvection, has been significantly improved compared to previous versions sequenced (Figure 1) from enrichments (Luedin et al., 2019a). The ability to maintain this species in laboratory conditions, albeit with phenotypic differences compared to environmental conditions (Di Nezio et al., 2024), has allowed for a significant increase in the quality of genome annotation. Most notably, the new assembly of *C. okenii* LaCa now comprises a single circular chromosome and an additional contig that may represent a plasmid. Most of the proteins contained in the smaller contig are also found in the previous annotation and in other Chromatiaceae species. This suggests that it could be a plasmid rather than an assembly artifact. The new assembly shows a completeness score of 93.51% and 0.28% contamination, compared to the older version's 72.75% completeness and 7.30% contamination. Average Nucleotide Identity (ANI) analysis showed very high concordance with the previously published LaCa genome 95.85%, but only 86.7% with the type strain *C. okenii* DSM 169 (GCF_016583745.1), originally from a lake in Ostrau, Germany. The low ANI with the type strain assembly can be explained by its high fragmentation (N50 88.9 kbp) and the relatively low coverage to which it was originally sequenced (12x). Despite this, 16S rRNA phylogenetic analysis showed high identity among the three assemblies as can be seen in (Supplementary Figure S2), suggesting they belong to the same species. The result is further confirmed by the conservation level of orthologous proteins (Figure 2A). This higher genomic resolution facilitates more accurate assessments of metabolic potential and provides a robust reference point for future comparative studies.

It is interesting to note that BUSCO scores show that 7.1% of the predicted single-copy orthologs of the Chromatiaceae family are missing from the assembly. Among the orthologs missing, we found the *dna*A gene, essential for genome replication initiation (Wegrzyn and Konieczny, 2023) and generally used as starting gene for bacterial assemblies (Mackiewicz et al., 2004). The absence of *dna*A in *C. okenii* and *Ca. Nitrosacidococcus tergens* suggests the existence of alternative replication strategies in the Chromatiaceae lineage. Several *dna*A-independent replication initiation mechanisms have been reported in cyanobacteria and Archaea (Ohbayashi et al., 2020; Dulermo, 2025); however, replication initiation was not investigated further in this work.

The genome of the “*invasive species*” *C. clathratiforme* (Decristophoris et al., 2009; Gregersen et al., 2009) was also finalized, representing the first for this species, which was previously named *Pelodictyon clathratiforme* and/or *Pelodictyon phaeoclathratiforme* (Imhoff, 2006), as also shown in the phylogenetic tree (Figure 2A). The genome of the second PSB from Lake Cadagno, T. syntrophicum, had already been sequenced in the past (Peduzzi et al., 2012; Luedin et al., 2018). The high similarity to the previous genome (>99%) sequenced 10 years ago (GCF_002813775.1) suggests that these microorganisms are not subject to major genetic changes outside their natural environment, even though the time period is certainly not adequate in an evolutionary context. The latest genome published, *C. phaeobacteroides*, is a new strain of the same species already sequenced in the past (GCF_000015125.1), found in the meromictic lake Blankvann in Norway (Davenport et al., 2010). Collectively, these improved assemblies strengthen the genomic framework for interpreting the ecological roles of sulfur bacteria in redox-stratified lakes and offer valuable new resources for evolutionary comparisons with strains isolated from other euxinic habitats.

### Bioconvection and transcriptional regulation

The improved quality of the *C. okenii* genome allowed us to further investigate the bioconvection process through transcriptomics. Previous studies showed that *C. okenii* mixes the BL only during the summer months (Sepúlveda Steiner et al., 2019, 2021; Storelli et al., 2025), typically from June to late August, while dominating photosynthetic activity compared to other PSB and GSB (Di Nezio et al., 2023). Bioconvection has been associated with longer daylight periods and higher light intensities characteristic of summer, whereas the earlier hypothesis that mixing requires a minimum bacterial density was ruled out (Sommer et al., 2017). Because reconstructing the natural environment, and thereby bioconvection, in laboratory conditions remains challenging, despite the availability of *C. okenii* LaCa pure cultures (Di Nezio et al., 2024), we opted to analyze the transcriptome of cultures incubated directly within the BL using dialysis bags.

Transcriptomic profiling of *C. okenii* cultures incubated *in situ* revealed marked differences between summer, a period characterized by active bioconvection, and early autumn, when bioconvection was no longer observed. During July, genes associated with cell proliferation and energy metabolism were strongly upregulated, consistent with enhanced growth and metabolic activity during periods when bioconvection occurs, potentially reflecting improved nutrient redistribution within the BL. Conversely, expression of genes involved in nitrogen fixation (*nifHDK, nifENB*, and associated cofactors) was significantly reduced, suggesting a potential reallocation of cellular resources away from nitrogen metabolism during periods of heightened motility and growth with high photosynthetic activity. Similar context-dependent adjustments have been reported at the ecosystem scale: Haynes and colleagues showed that complementary nitrogenases (V- and Fe-only) make substantial contributions to biological nitrogen fixation in environments with low CO_2_ fixation activity, emphasizing that diazotrophs flexibly deploy different strategies depending on resource availability (Haynes et al., 2022). Together, these findings highlight the metabolic flexibility of *C. okenii* LaCa, enabling it to balance growth, motility, and nitrogen fixation in response to shifting ecological conditions.

The transcriptomes obtained on the same day, one extracted during daylight and one at night, showed no significant differences in gene expression (data not shown). This suggests that the main metabolic functions remain largely unchanged between day and night, which may also explain why bioconvection does not stop in the absence of light (Sepúlveda Steiner et al., 2019, 2021). It is also interesting to note the difference compared to the other PSB *T. syntrophicum* Cad16^T^ sequenced in this study, where different proteomic regulation is observed, with proteins more present at night and/or during the day, suggesting possible regulation to promote CO_2_ fixation without light (Storelli et al., 2013, 2014; Berg et al., 2019). Importantly, the observed downregulation of *nif* genes during bioconvective periods should be interpreted as a correlative pattern rather than evidence of a direct causal link. Bioconvection co-occurs with multiple environmental changes, including increased light availability, temperature, and nutrient redistribution, any of which may independently or jointly influence nitrogen metabolism.

### Nitrogen fixation: *nif* genes and laboratory experiment

While the dialysis bag approach provided a valuable means to capture these *in situ* transcriptional dynamics, it is important to acknowledge potential artifacts related to confinement, altered gradients, and relatively low transcript coverage. Nevertheless, the reproducible expression patterns we observed underscore the critical role of physical processes, such as bioconvection, in shaping microbial gene regulation, consistent with recent *in situ* evidence that bioconvection influences microbial physiology and ecological interactions in Lake Cadagno (Di Nezio et al., 2023).

A central result of this study is that PSB possesses the complete genetic repertoire for diazotrophy and actively regulate *nif* gene expression *in situ*, thereby expanding a metabolic trait that was primarily associated with GSB in Lake Cadagno (Halm et al., 2009; Zimmermann et al., 2015). However, we observed substantial differences between PSB and GSB in terms of the organization of the *nif* operon (Figure 3B). For example, *nif* B is not located between E and N as in GSB, but 50 kb upstream of *nif* H. Furthermore, PSB, in addition to the genes necessary for nitrogen fixation -*nif* BEF (biosynthesis) and *nif* HDK (catalysis) are also equipped with a larger and more complex set of accessory *nif*-associated genes than GSB (Bennett et al., 2023).

Their ability to grow in the absence of any nitrogen source other than atmospheric nitrogen demonstrates that they can obtain the nitrogen required for survival and reproduction independently. Moreover, the lack of significant differences in growth between conditions with and without added nitrogen suggests that the N_2_ fixation process does not impose major physiological costs that would limit growth. The growth curves shown in Figure 5 indicate that both PSB *C. okenii* and *T. syntrophicum* were able to grow in the absence of added nitrogen compounds (without NH_4_Cl, red line, and with NaCl, green line) without evident growth limitations (Supplementary Table S7). Although minor differences in growth dynamics were detected, particularly for *T. syntrophicum*, which showed altered temporal trajectories under nitrogen-free conditions, these differences did not result in significantly higher final biomass. Thus, while growth dynamics were modulated by nitrogen availability and light intensity, overall growth performance remained comparable between nitrogen-amended and nitrogen-free treatments. This indicates that N_2_ fixation does not represent a substantial physiological constraint, but neither does it provide a clear growth benefit under these experimental conditions. In contrast, the reduced growth of the GSB control (*C. phaeobacteroides* 1VII D7) under nitrogen-free conditions warrants careful consideration. Although GSB are established diazotrophs, nitrogen fixation in these organisms is highly sensitive to culture conditions, including light intensity, sulfide availability, trace metal concentrations, and redox stability (Madigan, 1995). Suboptimal laboratory conditions may therefore suppress nitrogenase activity despite genetic potential, as reported by previous studies (Fay, 1992; Dixon and Kahn, 2004; Poza-Carrión et al., 2014).

The presence of nitrogen in the medium before and after bacterial growth was measured to essentially see two things: (i) whether combined nitrogen is preferentially assimilated when available, and (ii) whether nitrogen fixation results in detectable nitrogen release into the surrounding medium. In the first case, a decrease in ammonium present in the medium was observed, indicating a preferential uptake of readily available nitrogen sources over energetically costly fixation pathways. In the second case, ammonium concentrations remained at zero in nitrogen-free treatments, suggesting that fixed nitrogen was retained for cellular requirements rather than released into the medium. Importantly, the absence of ammonium accumulation does not imply a lack of nitrogen fixation, but rather indicates tight cellular regulation of nitrogen demand and assimilation.

These results corroborate a recent study that reported *nif* gene presence, expression, and *in situ* nitrogen fixation within the PSB of Lake Cadagno, particularly *C. okenii*, which alone consistently accounting for >80% of bulk N_2_ fixation (Philippi et al., 2021). Taken together, our findings support a model in which PSB are key contributors to nitrogen fixation *in situ*, yet laboratory growth assays reveal that this activity does not necessarily manifest as enhanced growth or nitrogen release under controlled conditions. Future studies using isotopic tracers (e.g., ^15^N_2_ incorporation) or acetylene reduction assays, ideally coupled with transcriptomic and proteomic monitoring of nitrogenase isoforms, will be essential to quantify rates and to determine the relative contributions of PSB, GSB, and other diazotrophic populations.

## Conclusions

The combined genomic, transcriptomic, and physiological results provide a coherent picture of PSB adaptation to the euxinic zone of Lake Cadagno. The high-quality genome assemblies of *C. okenii* LaCa and *T. syntrophicum* Cad16^T^ revealed the presence of complete nitrogenase gene clusters, including the structural components *nifHDK* and the assembly/activation genes *nifENB* and associated cofactors, establishing the genetic basis for nitrogen fixation. It is interesting to note that PSBs, compared to the more famous GSBs nitrogen fixers, have a greater number of *nif* genes (Figure 3A) as well as better growth in the absence of nitrogen sources (Figure 4, green lines). Transcriptomic profiling then showed that expression of these genes was actively regulated *in situ*, with *nifHDK* and *nifENB* transcripts significantly downregulated during periods of strong bioconvection and photosynthetic activity, consistent with a reallocation of resources away from nitrogen fixation when nutrient mixing favored rapid growth and motility (Figure 3). Finally, laboratory experiments demonstrated that *C. okenii* LaCa and *T. syntrophicum* Cad16^T^ are capable of sustaining effective growth in the presence or absence of a nitrogen source, a behavior consistent with the regulated expression of nitrogenase genes and the potential for diazotrophic metabolism under conditions of limited nitrogen availability.

Together, these results integrate genomic potential, transcriptional regulation, and physiological observations, highlighting how PSB coordinate multiple levels of metabolic control under euxinic conditions. This work reinforces the value of meromictic lakes as “*modern analogs*” and provides a foundation for interpreting the ecological strategies of ancient microbial phototrophs within modern biogeochemical frameworks.

## Data Availability

The data presented in this study are publicly available. The data can be found at: https://www.ebi.ac.uk/ena/browser/home, accession PRJEB110913.
