# Supplementary material for: Genomics, transcriptomics, and laboratory experiments link bioconvection to nitrogen fixation
Source: Front Microbiol. 2026 Apr 2;17:1760084. doi: 10.3389/fmicb.2026.1760084 (PMC13116554; doi:10.3389/fmicb.2026.1760084)
Supplement: Supplementary file 1 [file Data_Sheet_1.docx]

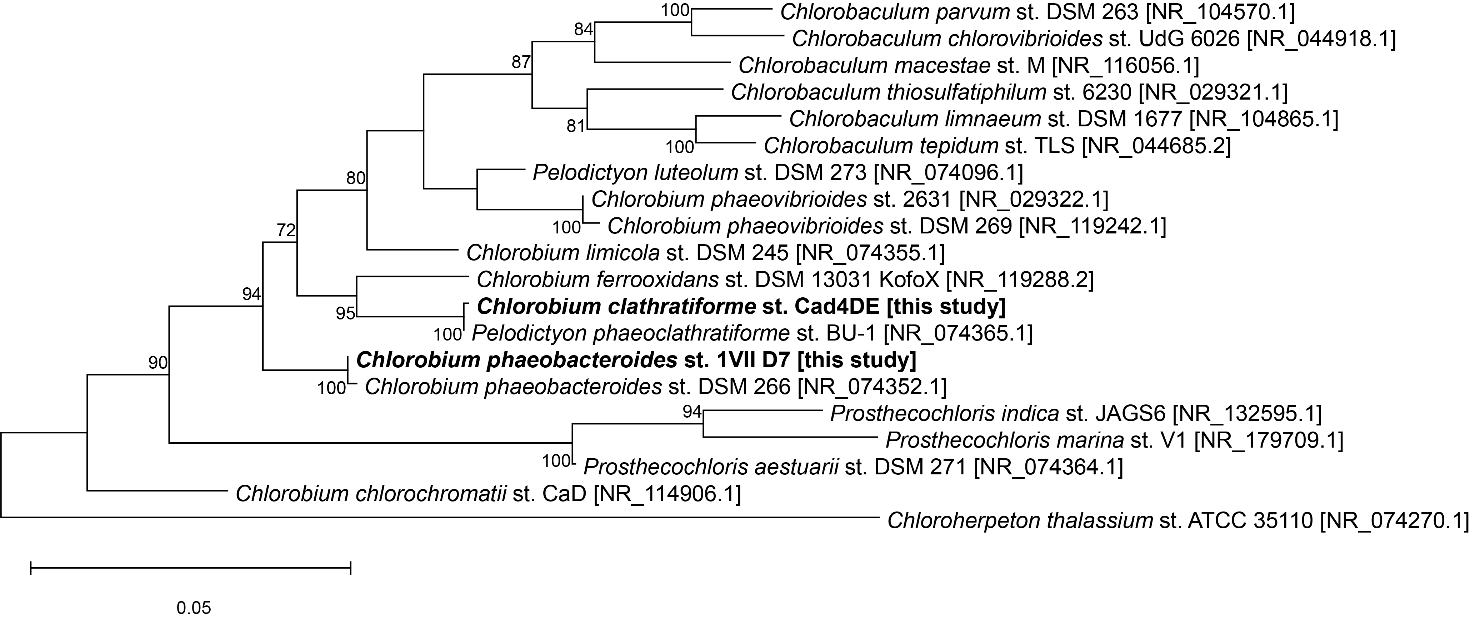
***Figure S1. Phylogenetic relationship of the two Green Sulfur Bacteria from this study within all publicly available 16S rRNA of closely related species (Chlorobiales order)****. The maximum likelihood consensus tree was constructed from one hundred single-copy orthologs randomly selected. Bootstrap support values are shown for nodes with support higher than 70%.*

***
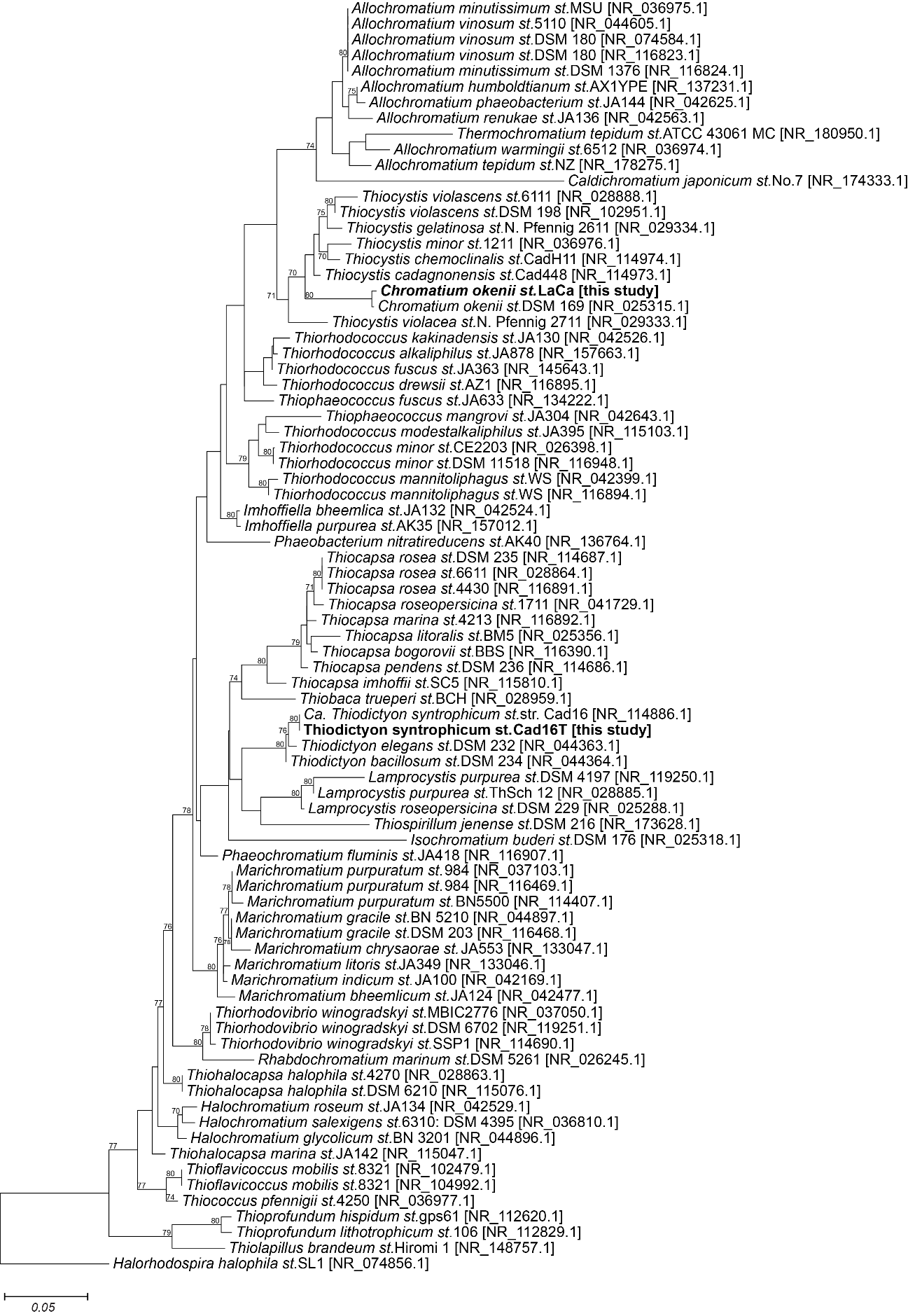
Figure S2. Phylogenetic relationship of the two Purple Sulfur Bacteria from this study within all publicly available 16S rRNA of closely related species (Chromatiales order)****. The maximum likelihood consensus tree was constructed from one hundred single-copy orthologs randomly selected. Bootstrap support values are shown for nodes with support higher than 70%. Abbreviations: Candidatus (Ca.), strain (st.).*

***
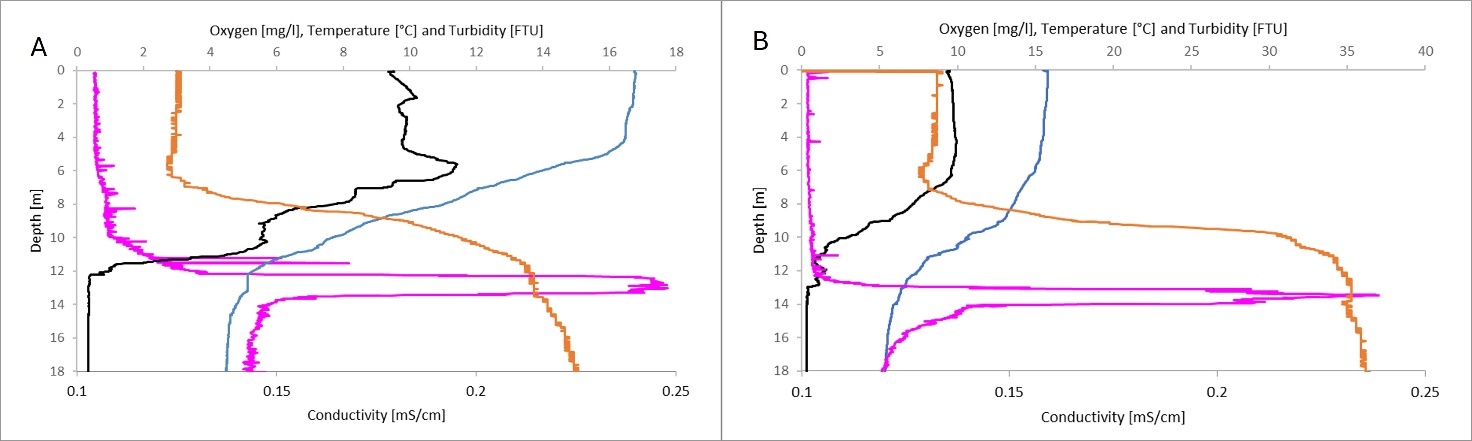
***

***Figure S3. The water column profiles measured during the transcriptomic analyses in July (July 16, 2020) and September (September 17, 2020).*** *Profile of the water column of Lake Cadagno with curves showing temperature (blue), conductivity (orange), oxygen (black), and turbidity (pink) on A) July 16, 2020, and B) September 17, 2020.*


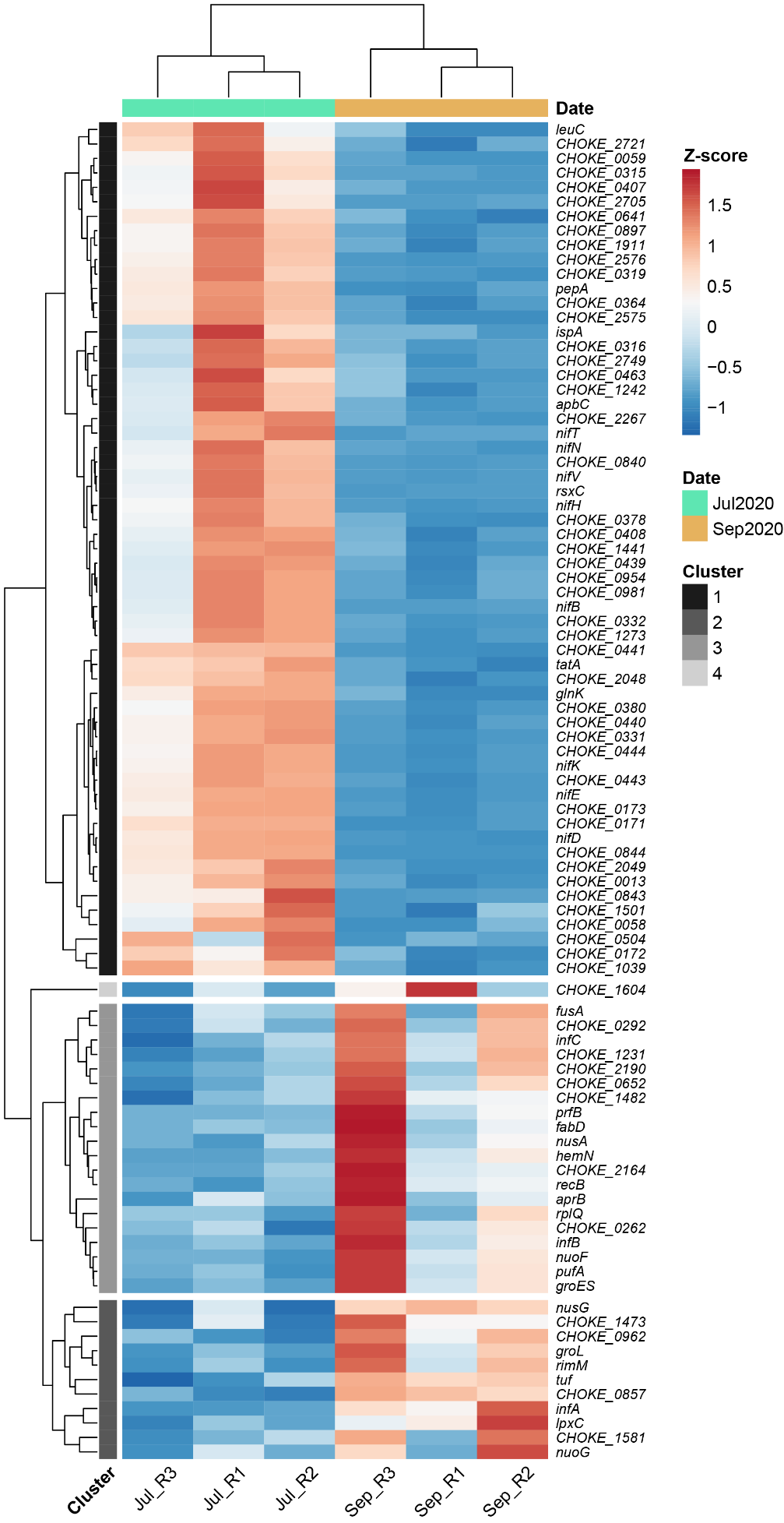


***Figure S4. Heatmap of* Chromatium okenii *normalized gene expression (row-wise z-scores) in July and September of 2020.*** *Rows and columns were grouped using hierarchical clustering. Gene IDs with the CH OKE prefix correspond to predicted genes. Only differentially expressed genes (|log2FC| > 1, FDR < 0.05) are shown.*
